# Micro-Scale Surface Patterning of Titanium Dental Implants by Anodization in the Presence of Modifying Salts

**DOI:** 10.3390/ma12111753

**Published:** 2019-05-30

**Authors:** Gaetano Marenzi, Gianrico Spagnuolo, Josè Camilla Sammartino, Roberta Gasparro, Alberto Rebaudi, Marco Salerno

**Affiliations:** 1 Department of Neurosciences, Reproductive and Odontostomatological Sciences, University of Naples “Federico II”, Via Pansini 5, 80131 Naples, Italy; gaetano.marenzi@gmail.com (G.M.); roberta.gasparro@unina.it (R.G.); 2 Institute of Dentistry, I. M. Sechenov First Moscow State Medical University, 119146 Moscow, Russia; 3 Department of Biology and Biotechnology “L. Spallanzani”, University of Pavia, Via Ferrata 1, 27100 Pavia, Italy; jose.sammartino@iusspavia.it; 4 Rebaudi Dental Office, Piazza della Vittoria 8, 16121 Genova, Italy; alberto.rebaudi@gmail.com; 5 Materials Characterization Facility, Istituto Italiano di Tecnologia, via Morego 30, 16163 Genova, Italy; marco.salerno@iit.it

**Keywords:** titanium alloy, dental implant, anodization, nanopores, surface treatment, micro-morphology, titania, calcium ions, potassium ions

## Abstract

The bone-implant interface influences peri-implant bone healing and osseointegration. Among various nano-engineering techniques used for titanium surface modification, anodization is a simple, high-throughput and low-cost process, resulting in a nanoporous oxide coating which can promote osseointegration and impart antimicrobial and immunomodulatory properties. We anodized rounded tip dental implants of commercial grade titanium in aqueous phosphoric acid modified with calcium and potassium acetate, and characterized the resulting surface morphology and composition with scanning electron microscopy and energy dispersive spectrometry. The appearance of nanopores on these implants confirmed successful nanoscale morphology modification. Additionally, the metal cations of the used salts were incorporated into the porous coating together with phosphate, which can be convenient for osseointegration. The proposed method for surface nanostructuring of titanium alloy could allow for fabrication of dental implants with improved biocompatibility in the next stage of research.

## 1. Introduction

In implant dentistry, titanium (Ti) and its alloys are the most commonly used materials due to their biocompatibility, mechanical characteristics and chemical stability. As with most metals, in wet or even ambient air environment, Ti is coated with a thin layer of native oxide, namely TiO_2_ (titania). This oxide coating is the surface that establishes a direct contact between the dental implant and the surrounding alveolar bone, a process known as osseointegration [1,2]. Many studies have reported that the nature of bone–implant interface can influence peri-implant bone healing and the osseointegration process [3,4,5,6,7]. The first-generation osseointegrated surfaces were produced by industrial machining or turning of a bulk titanium implant, which led to a polished or minimally rough surface with some residual periodic microgrooves [7,8,9,10]. To improve the clinical success, further processing was developed to increase the micro-roughness of the surface by using either chemical (acid-etching) or mechanical treatment (grit-blasting), or a combination of the two [11,12]. A rough implant surface not only ensures improved bone anchorage when compared to a smooth implant surface, but also promotes mesenchymal cell differentiation toward osteoblastic phenotype [13,14]. However, optimal roughness values and surface morphology pattern are still controversial and need to be more clearly defined [2,15]. One current hypothesis is that to augment implant surface bioactivity and promote both early integration and long-term success, dental implant surface should carry both macro, micro and nanoscale features and related roughness [16,17,18]. While the macroscale shape depends on machining, and the microscale roughness can be successfully obtained by etching or sand-blasting, it has been shown that anodization is an effective means to obtain nanoroughness, in the form of anodic porous titania (APT). This material has the ability to modulate cellular responses and facilitate local therapeutic release [19]. Typically, the anodization of titanium is carried out in aqueous/organic electrolyte containing fluoride ions, by applying a constant voltage or current to the electrochemical cell [20,21]. The resulting nanopores diameter, length and smoothness can be controlled by varying the electrochemical parameters [13,19,20,21,22,23,24,25]. Several in vitro and in vivo studies have established that APT is capable of promoting osseointegration and soft-tissue integration, as well as imparting antimicrobial and immunomodulatory properties, compared with conventional macro- or micro-rough implant surfaces [26,27,28,29]. Anodization is a simple, cost-effective and tailorable approach [20,26] that can promote micrometer-scale thickening and ionic impregnation of the titania layer, whereas the collapse of the surface material results in porous structures and associated micro- or nano-topography [10]. Implant surface titania can exist in three crystallographic structures: anatase, brookite and rutile. Sul et al. [30] showed that the type of crystalline oxide formed on the implant surface during the micro-arc oxidation processing changes with the treatment method, temperature and thermodynamic conditions [26]. Electrochemically formed oxide films on titanium can be either amorphous or crystalline, depending on the anodic potential and electrolyte used. Higher temperatures induce rutile formation, while lower ones induce anatase formation. It has been shown that dental implants with anatase phase titania on the surface show better biocompatibility performance than those that exhibit a rutile phase structure [31].

The aim of this research was to combine the fabrication of the nanomorphology on the surface of dental implants coated with ATP, with the incorporation of calcium (Ca) and potassium (K) into the porous coating, with the perspective of future improvement of the bioactivity of conventional dental implants.

## 2. Materials and Methods

### 2.1. Implants and Chemicals Used

**Figure 1:**
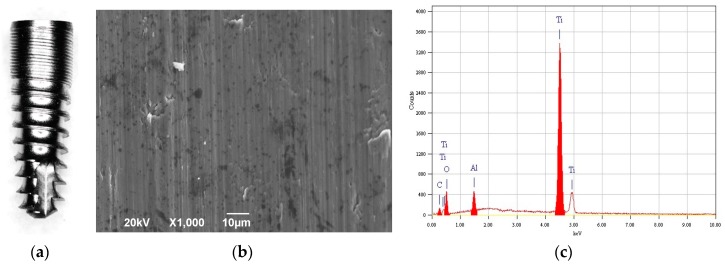
(**a**) Optical micrograph of the type of implant investigate in this study; (**b**) representative SEM micrograph of M implant surface, and (**c**) corresponding EDS spectrum.

Commercially pure titanium (grade 4) dental implants ISO3 (Stark, Monaco, France) were used (see Figure 1a). These implants have conical macromorphology with rounded apical tip, single thread and 4.1 mm diameter and 10 mm length. Two implants were kept in machined form only (group M), without any surface roughening treatment (and subsequently wet, one in each of the two aqueous salt solutions, of Ca or K); two implants were sandblasted (group S) according to the standard manufacturer production (and subsequently wet, one in each of the two aqueous salt solutions, of Ca or K); six implants were sandblasted and anodized (SA) according to different conditions (one for each combination of three different concentrations and two different salts, as explained in Section 2.2.). The implants were sterilized and sealed in plastic boxes to minimize possible contamination prior to anodization and subsequent analysis, and were handled with clean tweezers and gloves.

For the functionalization with Ca and K, we used acetate salts Ca(CH_3_CO_2_)_2_ and (K(C_2_H_3_O_2_)) (Sigma Aldrich, Milan, Italy), respectively. For dipping the implants in the liquid solutions, we used the metallic screw available for prosthetic connection which fit the inner base cavity of the implants. The two implants each of groups M and S were kept for 1 min at room temperature in the aqueous solution of Ca or K acetate at intermediate concentration only (1 M).

### 2.2. Anodization

The six implants of group SA were anodized after sandblasting. The anodization was carried out in aqueous solution 1.5 M of phosphoric acid (H_3_PO_4_), and with addition of either Ca or K acetate salt at different concentrations of 0.5, 1 and 1.5 M. The counter electrode (negatively biased cathode) was an inert Pt mesh basket (~2.5 cm diameter), placed under the implant at a distance of ~1 cm from its lowest point (i.e., the tip). The source of electrical energy was high power direct current supply (N5751A, Agilent Technologies, Santa Clara, CA, USA), connected for both control and output to a laptop computer. A visual basic macro allowed for the collection of both current (i) and voltage (V) data from the circuit. The anodization proceeded for 1 min in mixed potentiostatic-galvanostatic mode, by setting limiting values in both voltage and current, to 150 V and 0.2 A, respectively. To keep the setup as simple as possible, the process was carried out starting from room temperature without any cooling system, in a simple, fully-filled 100 mL glass beaker.

A full identification of the implants in the form of short codes is reported in Table 1. There, where different concentrations of the salts are used, “−” and “+” stand for the lowest and highest concentration, respectively, with no sign for the intermediate one.

### 2.3. Imaging and Chemical Analysis

All the implants were mounted on aluminium (Al) stubs by means of silver paint, and were sputter-coated with a ~10 nm thick layer of carbon (C), before inserting into the SEM chamber. We used a SEM instrument (JSM-6490LA, JEOL, Akishima, Japan), equipped with energy dispersive spectroscopy (EDS) add-on by the same manufacturer for the chemical identification of the surface coating composition, working with primary electron beam energy of 20 keV. For imaging, the instrument was operated at aperture 2 and spot size 30, while for EDS we used aperture 3 and spot size 60 to maximize the signal. The surface inspected by SEM was selected to always be in the thread valleys, to appear as flat as possible in the background. However, the treatment should be conformal and uniform all over the surface as a result of the wetting by the liquid electrolyte. For the EDS analysis and elemental image overlay, we used the OEM software tool Analysis Station version 3, 8, 0, 59 (JEOL Engineering Co., Ltd., Akishima, Japan), including the JED Series Mapping Program.

### 2.4. Statistical Analysis

For each group, represented by a single implant with specific treatment, the SEM images and the corresponding EDS analysis were taken in four different locations on the treated surface, and the quantitative chemical composition content of interest (Ca or K) was averaged. When comparing the above contents among different treatments, analysis of variance was carried out with the same program OriginPro9 as used for plotting the bar graphs, and the differences were analyzed for statistical significance (at least *p* < 0.05) under pair test comparisons with the Tukey criterion.

## 3. Results

The typical lay of implant M surface is shown in the SEM image of Figure 1b, and concurrently Figure 1c reports the chemical composition of the same region as assessed by EDS. Obviously, the Ti from the implant body represents the majority of the contents. Additionally, one can find O from the native oxide and C from the coating. Al is also present, as observed previously [16], which is probably due to contamination in the machining process.

**Figure 2:**
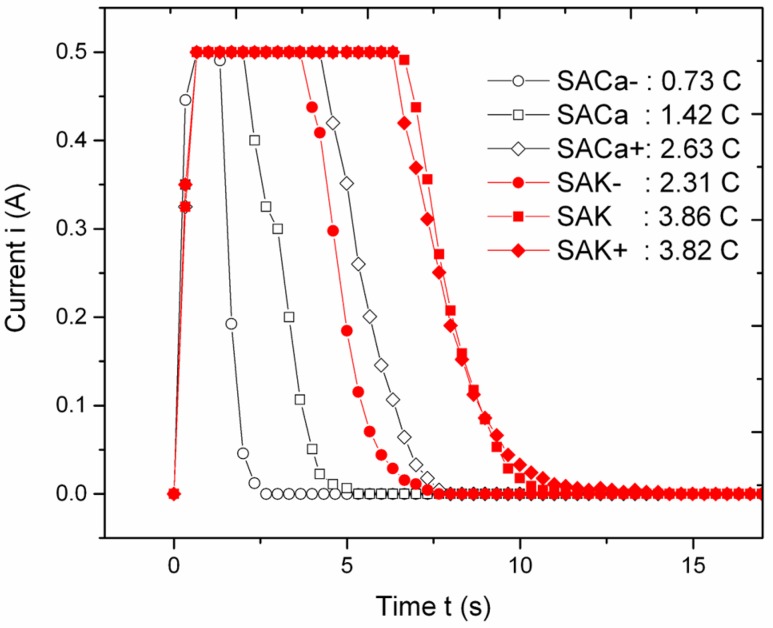
Chrono-amperometric profiles of implants SA during anodization. In the legend, the total area (i.e., passed charge) is also reported, for each profile.

In Figure 2, the chrono-amperometric plots for anodization of SA implants have been reported. Since the anodization was carried out by setting limiting values in both current and voltage, it was a so-called mixed mode, i.e., neither completely potentiostatic nor galvanostatic. From Figure 2, it appears that in all cases the current initially increased until reaching the limiting value of 0.5 A, then stayed constant for a few seconds, and finally decreased, so that it was zero after 12 s for all processes. During the time that the current was constant, the voltage (not shown) was still increasing to keep the current constant during the oxide growth. Finally, when the current was zero, the voltage was set to the limiting value of 150 V, however it was obviously ineffective, due to the already present oxide. Thus, for most of the time, the process was potentiostatic, with the voltage driving the pore size, along with the acid concentration [30]. However, the most effective time period was actually galvanostatic.

The current profiles in Figure 2 are rather different in underlying area, which is the total charge passed, and can be tentatively distinguished according to the metal additive in the electrolyte. The curves for Ca are grouped around central charge values close to those (~1.2 C) obtained previously for magnesium (Mg) [16]; this is consistent with the expected similarity in behavior between the two bivalent ions, Ca^++^ and Mg^++^. Within this group, the high-current duration (and thus the passed charge) is ordered according to the increasing availability of the respective metal ion, i.e., the salt concentration, even if it scales sub-linearly with the latter. The group of current profiles for K presents higher average passed charge levels than for Ca, and more intermixed (as those for 1 and 1.5 M salt are overlapped). While the K^+^ charge is lower, the total passed charge being higher may be ascribed to the lower mobility of Ca^++^ in water vs. K^+^, due to the higher solvation number of water molecules.

**Figure 3:**
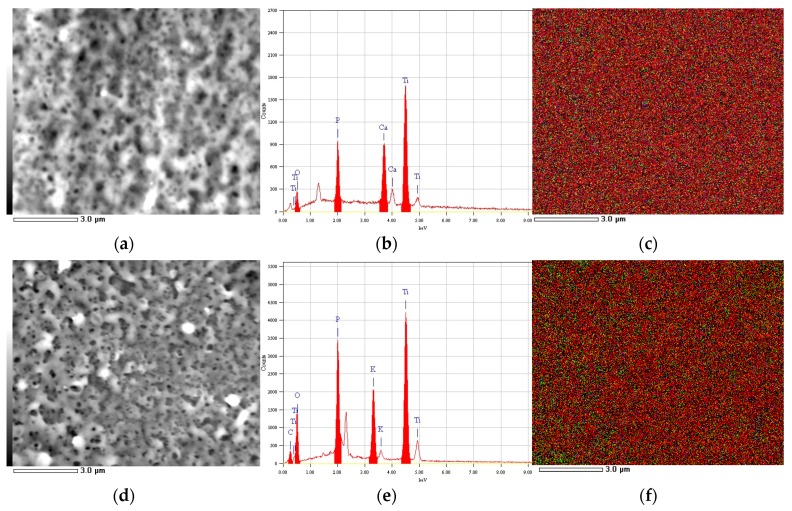
SEM micrographs (**a**,**d**), EDS spectra (**b**,**e**) and overlaid color maps of elements P (red pixels), O (green pixels), and either Ca (**c**) or K (**f**) (blue pixels), respectively; (for the representative case of 1 M salt concentration).

Despite the different ions in the electrolytic solution and the different charge passed, the top surface of most implants appeared similar, with apparent pore size and spacing of the order of ~0.3 µm and ~1 µm. This emerges from Figure 3a,d, where the typical nanoporosity of APT is shown for the SACa and SAK groups at intermediate salt concentration (1 M). In the panels on the right to the pictures (i.e., Figure 3b,e) the EDS spectra of the respective areas are reported. Finally, on the rightmost side of Figure 3 (i.e., in Figure 3c,f), the maps showing the localization of different elements, P (in red), O (in green) and Ca or K (in blue) appear, in overlaid colors. The uniform distribution of each color demonstrates that the oxide, as well as the biofunctionalization with P and the additional metal cation (either Ca^++^ or K^+^), are all homogeneous throughout the implant surface.

The P contamination appearing at a concentration of ~11 at% in both Figure 3b,e, is due to the phosphate anions PO_4_^− −^ entrapped in the porous oxide during its growth [18]. In both Figure 3b,e, the presence of Ti oxide grown on the surface is confirmed, as the O contents significantly increased with respect to Figure 1c from ~3 to at least ~7 at%. It has been shown previously that the as anodized APT should be mainly anatase, which should be convenient for osseointegration [16]. The difference in total passed charge observed in Figure 2 may thus be due not only to the metal ions migrating to the cathode, but also to the ions contributing to oxide growth, i.e., be associated with increased anodization efficiency. Overall, it appears that there is more green (i.e., O) for the case of K functionalization (Figure 3d) as compared to Ca (on average, ~14 vs. ~7 at%). This makes us think that the higher current for anodizations in the presence of K^+^ is actually due to oxide growth, rather than the transport of K.

**Figure 4:**
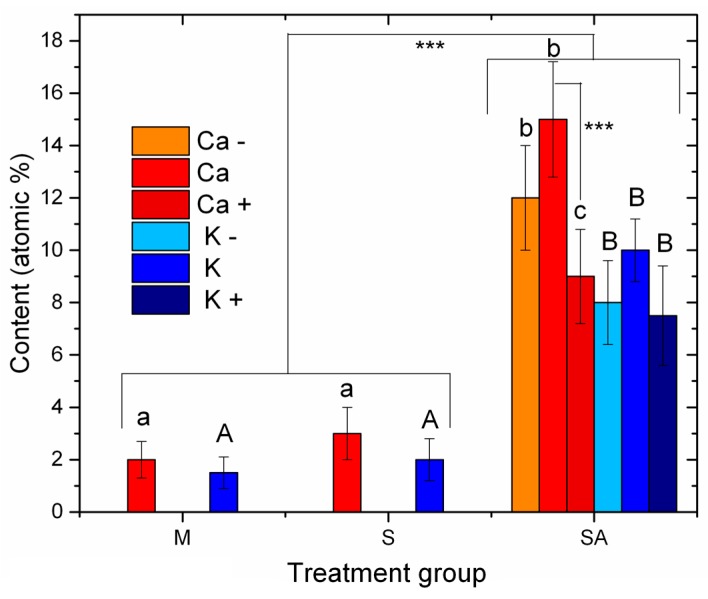
Quantitative composition data of the metal from the salt added to the anodization electrolyte, extracted from the EDS spectra of all the different implants treated with salt solutions. The statistically different groups have been identified by different letters, lowercase for the Ca (a, b, c) and uppercase for the K (A, B).

In Figure 3b,e, the presence of the metal ion from the respective salt added to the electrolyte is confirmed. From the EDS spectra of all the implants treated with the metal salts, we extracted the atomic percentage contents of both Ca and K and plotted these data in Figure 4. The bar heights represent the mean values (N = 4), and the error bars are ± one standard deviations. The letters represent the statistically significant different groups, which were tested for both *p* < 0.05 (*) and, in the latter case, additionally for *p* < 0.001 (***). Small letters have been used for the Ca and uppercase letters for the K groups.

In Figure 4, it mainly makes sense to compare different treatments done with the same metal salt X (either Ca or K), that is, MX, SA, SAX−, SAX and SAX+. It appears that the M and S implants were in all cases much lower in metal content (*p* < 0.001) than all of the SA implants. Obviously, anodizing in the presence of the ions allowed for either incorporation in the oxide walls or loading into the deep pores, which made the sonication mostly ineffective in removing the loaded ions. At the time of our former work with Mg, while we had the impression that the higher concentration (1.5 M) allowed for better surface coverage than the lower one (0.5 M), we commented that it could perhaps also depend on aggregation. This would eventually cause the presence of loosely bonded material on the surface, possibly giving rise to weak interface bonding during the early stages of implantation, i.e., result in poor primary stability. Whereas here as well, we did not prove actual incorporation of the respective metal ions in depth into the nanoporous oxide coating. After the sonication step we may be more confident that the remaining ions are not loosely bonded to the surface only, which will hopefully prevent the above-mentioned issue. Consistently, the EDS elemental maps describe a uniform distribution, possibly due also to the presence of disordered, branching pores under the top surface.

For the S implants, the means of metal content were slightly higher than those of the M implants. One could think that the increased roughness originating from the sandblasting facilitated adsorption of the metal ion from the salt into recessed regions that were less efficiently cleaned also with sonication. However, the differences were not statistically significant.

Within the SA groups, the only statistically significant difference (*p* < 0.001) appeared for SACa vs. SACa+.

## 4. Discussion

Many research efforts have been directed towards improving the bone/implant interface with the aim of accelerating bone healing and improving bone anchorage to the bone. Anodization is a recently introduced surface modification method to modify the structures and chemical properties of anodic film of titanium by controlling the electrochemical parameters, such as electrolyte composition and concentration, applied potential or current temperature. The prepared porous titania nanostructures appear to be able to improve apatite formation [32,33,34].

Despite the numerous advancements in the field of anodization into the current dental implant market, research gaps include the lack of fabrication optimization, performed on a substrate of conventional implant micro-topography and inadequate mechanical stability [20,26]. Dental implants are not flat surfaces but 3D objects with curved surfaces, which can increase the chance of anodic film cracks and delamination, owing to greater internal stress, volume expansion and the presence of so-called “weak spots” [35,36]. These conditions may compromise implant stability and lead to toxicity and complete implant failure. The results of this research showed that a single process was adequate to assure a well-adhered and crack-free porous titania layer on implant surface. Another problem regarding the clinical use of implants modified by nanocomposites coating was recently reported: autoclaving of titania nanotube coating leads to the destruction of its architecture changing their mechanical biointegration properties [9].

Many authors evidence how the surface anodized treatment assure the opportunity to incorporate other ions in to implant surface. With this approach, the dental implant interface was improved chemically by incorporating inorganic phases such as calcium phosphate, Mg and Zn favoring stimulation of bone regeneration and the biochemical interlocking between bone matrix, proteins and fixture surface [7]. It is reported that the presence of divalent cations chemistries, CaTiO_3_ or MgTiO_3_, together with thin ≤4 μm titanium oxide causes rapid and strong interaction of the implant with bone via biochemical bonding at the bone-implant interface [26]. In the implant examined in the former work, an incorporation of Mg ions into the porous coating were attempted. Ca ions were selected not for promoting the osseointegration process, but as essential to all living cells for its interaction with polyphosphate compound such as ATP, DNA, and RNA. The presence of Ca in the anodized implant surfaces, as evidenced by SEM and EDS, seemed to be in relation to the ATP formation, as the Ca was coated less efficiently in the examined implant sites where the ATP did not form. However, Ca overlay result in apparent clogging of the APT pores; this may change the desired nanostructure patterned by the anodization. It is therefore important and not trivial to try to load bioactive materials into APT, as it can promote the development of a new generation of titanium implants with improved biocompatibility. We addressed this task here, focusing on Ca and K as the modifying additives.

In a former work we investigated the possible incorporation of Mg, in addition to P coming from the phosphoric acid used for the anodization. However, there are several other metal ions that can be expected to be beneficial in a number of physiologic processes, and thus potentially enabling enhanced osseointegration at the implant site. Among them, Ca couples conveniently with phosphate, together making a material similar to the bone contents itself (e.g., hydroxyapatite). Additionally, K and sodium (Na) may also play an important role [37]. However, while Na may be more readily accessible from the diet, K in the intracellular fluid would eventually be less easy to retrieve and thus more key to be released from the implant itself. Therefore, we decided to test with both Ca and K. The latter would be commonly available from KCl, but it is safer to have it free of chlorine We therefore used K acetate for both Ca and K cations, as acetate is probably one of the most biocompatible anions.

Three different concentrations of the metal acetate salt were tested during anodization, to possibly find an optimum amount of metal functionalization. However, to limit the number of implants used in the control cases of the implant surfaces not anodized, but only dipped in the salt solution, we limited to the central (intermediate) concentration only (i.e., 1 M) in those two cases. Whereas we had no active cooling system operating during the process in this experiment, the time of the passing current was so short that no major heating of the solution, and thus of the implants, occurred during the process. In the end, the measured temperature was ~35 °C in all cases. Also, compared to the former work [18], no preliminary cathodization, i.e., passing current under inverted polarity, was carried out here, as in that case no apparent difference in the surface nanostructure had been observed. All these simplifications confirm that anodization is a simple and inexpensive means of nanopatterning the Ti surface.

According to SEM morphology, no apparently relevant difference was observed among the different locations explored on each implant (N = 4). However, we observed (images not shown) that at the highest concentration of K salt, the pores were lesser in number and locally absent in some regions (images not shown). We assume that in those cases the excess of current due to the metal ions decreased the efficiency of the oxide ion diffusion current, limiting not only the thickness of the oxide but even the local growth of the columnar pores. A deeper understanding of this side-effect is beyond the scope of the present work. Here, we rather intended to demonstrate that the surface of the treated implant may present the typical pores of APT (see Figure 3b,e) with additional presence of the desired metal ions.

## 5. Conclusions

It is confirmed that anodization in acid electrolyte provides a conformal coating with pores also formed on curved surfaces of commercial grade Ti, even in the absence of cathodic pretreatment step, and is thus a viable inexpensive approach for the nanopatterning of dental implant surfaces. By anodizing in phosphoric acid, the phosphate from the dissolved acid anions is incorporated into the grown nanoporous oxide, and also, upon the addition of extra metal cations of potential biological interest, these can be incorporated too, providing a potential biofunctionalization of the APT surface. The next step in our research will be the assessment of the functionality of the prepared coatings by in vitro experiments with osteoblast cell cultures and possibly by in vivo experiments in animal models.

## Figures and Tables

**Table 1:** The groups of dental implants characterized.

Treatment	Metal of the Added Salt	Salt Concentration (M)	Implant ID
Machined only (M)	Ca	1	MCa
	K		MK
Sandblasted (S)	Ca		SCa
	K		SK
Sandblasted + Anodized (SA)	Ca	0.5	SACa−
		1	SACa
		1.5	SACa+
	K	0.5	SAK−
		1	SAK
		1.5	SAK+
